# Supplementary figures and images for: Mycobacterium spp. exposure, childhood vaccinations, and early childhood brain and CNS cancers
Source: Front Immunol. 2025 Jan 24;16:1497436. doi: 10.3389/fimmu.2025.1497436 (PMC11815319; doi:10.3389/fimmu.2025.1497436)

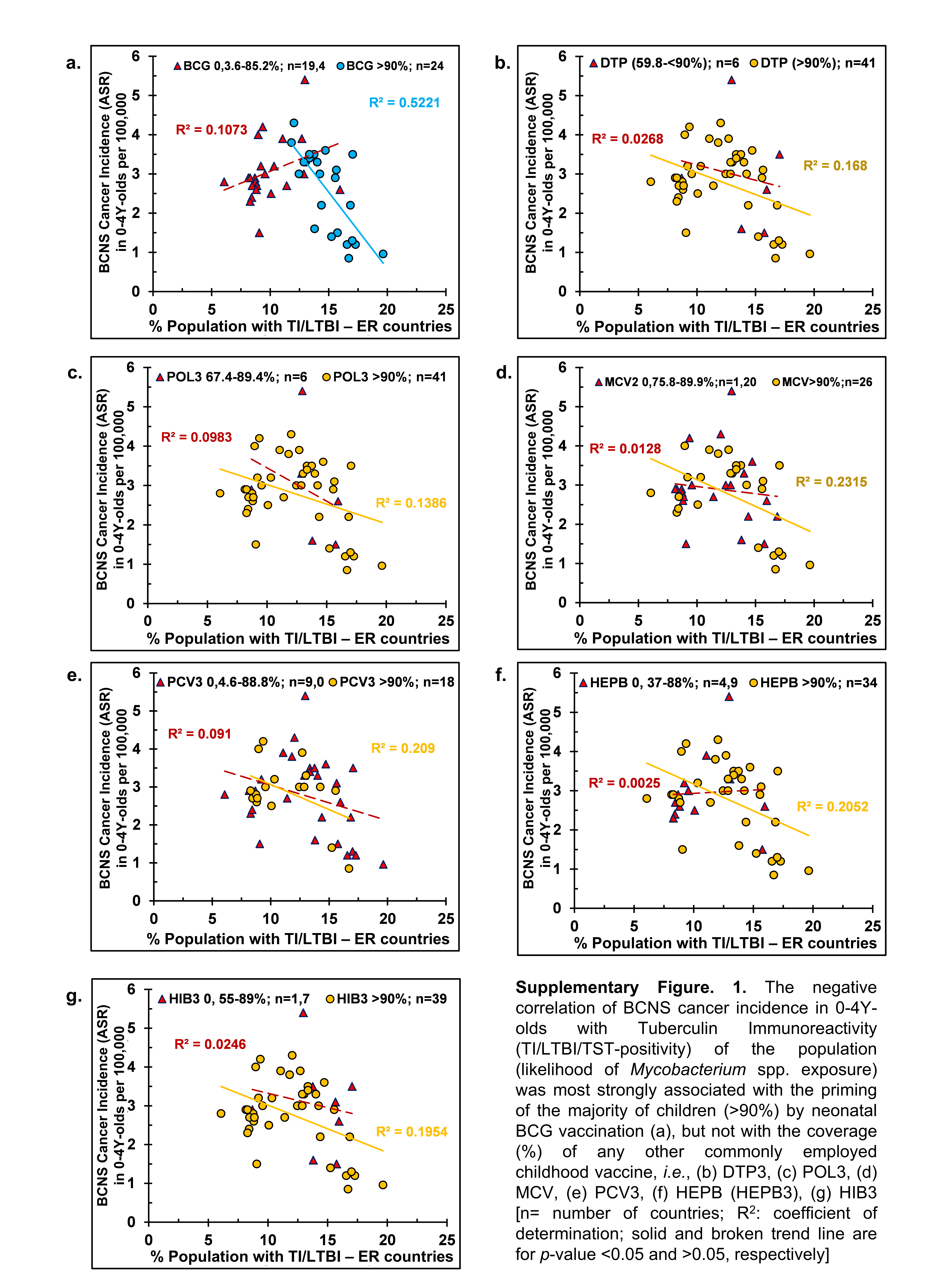

Supplement: Supplementary Figure 1 — The negative correlation of BCNS cancer incidence in 0-4Y-olds with Tuberculin Immunoreactivity (TI/LTBI/TST-positivity) of the population (likelihood of Mycobacterium spp. exposure) was most strongly associated with the priming of the majority of children (>90%) by neonatal BCG vaccination (A), but not with the coverage (%) of any other commonly employed childhood vaccine, i.e., (B) DTP3, (C) POL3, (D) MCV, (E)PCV3, (F) HEPB (HEPB3), (G) HIB3 [n= number of countries; R2: coefficient of determination; solid and broken trend line are for p-value <0.05 and >0.05, respectively]. [file Image1.tif]
